# Psoas Abscess in Spinal Tuberculosis

**DOI:** 10.4269/ajtmh.25-0088

**Published:** 2025-05-06

**Authors:** Raktim Swarnakar, Shiv Lal Yadav

**Affiliations:** ^1^ Department of Physical Medicine and Rehabilitation, National Cancer Institute (NCI-AIIMS), Jhajjar Campus, All India Institute of Medical Sciences (AIIMS), New Delhi, India;; ^2^ Department of Physical Medicine and Rehabilitation, All India Institute of Medical Sciences (AIIMS), New Delhi, India

**Figure 1.**
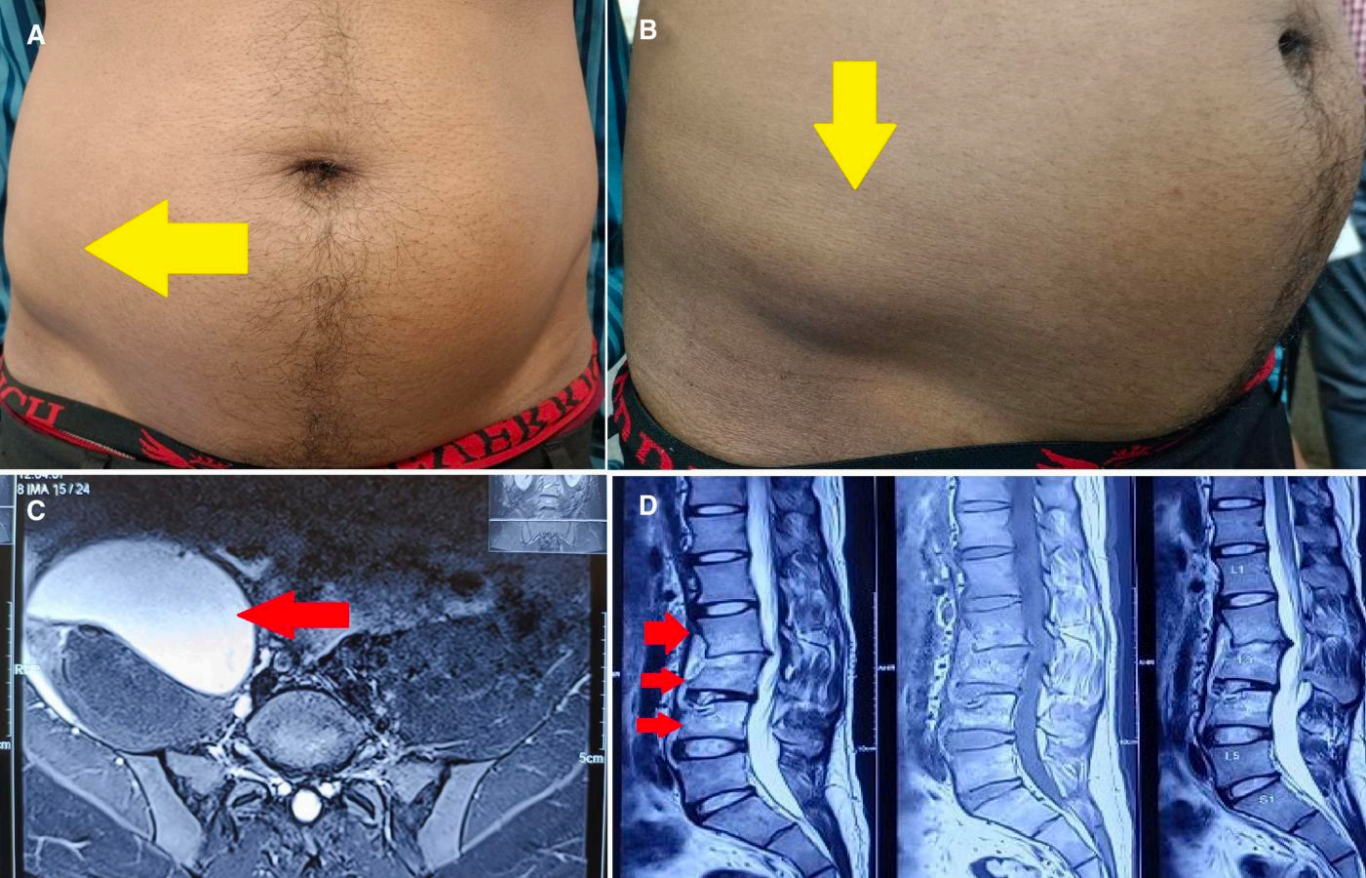
Psoas abscess in spinal tuberculosis. (**A** and **B**) Clinical image showing fullness of right lumbar and right iliac area (yellow arrow in front view and right lateral oblique view). (**C**) Axial magnetic resonance imaging (MRI) showing right large psoas abscess (red arrow). (**D**) Sagittal MRI showing tubercular involvement of multiple lumbar vertebras (L2, L3, L4) (red arrows).

**Figure 2.**
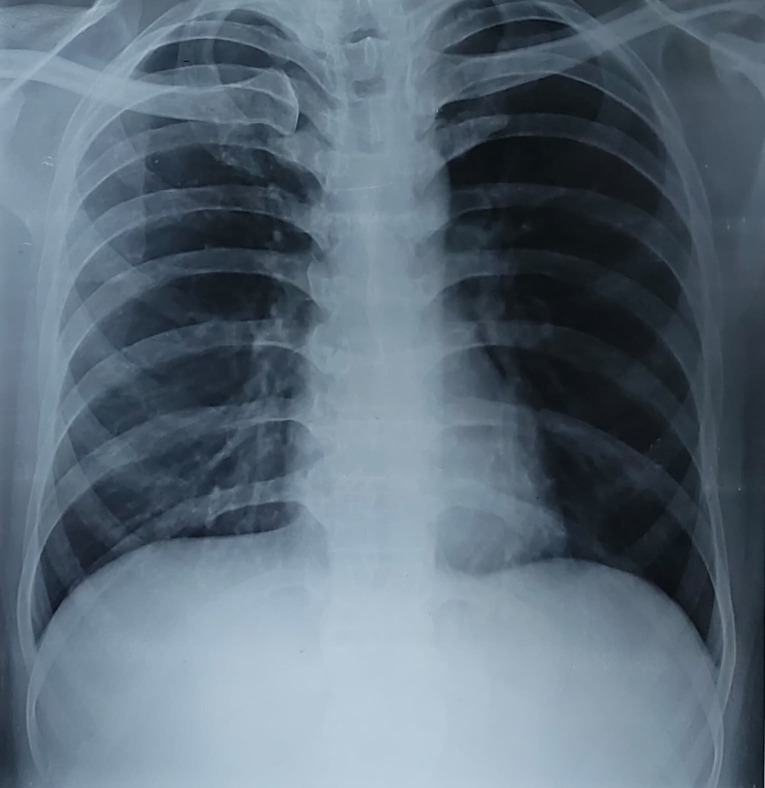
Chest X-ray (posterior–anterior view) showing no evidence of active or old tuberculosis.

A 26-year-old man from Bihar, India, presented with low back pain and low-grade evening rise of temperature for 1 month. He also had history of weight loss and loss of appetite. On examination, temperature was mildly raised, fullness was noted over the right lumbar and right iliac regions (Figure 1A and B), but no neurological deficits were present. Magnetic resonance imaging (MRI) revealed a large psoas abscess (Figure 1C) and involvement of multiple lumbar vertebrae (L2, L3, L4) (Figure 1D).^1^^,^^2^ Serum C-reactive protein (CRP) was 55 mg/dL (reference value <5 mg/dL). Total leukocyte counts and other routine blood tests were normal. Chest X-ray was normal (Figure 2). Ultrasound-guided pigtail catheter drainage was performed, and GeneXpert™ Mycobacterium tuberculosis (TB)/resistance to rifampicin was positive; rifampin (R) resistance was not detected.^3^ No spinal stabilization surgical intervention was needed. The patient was started on anti-TB drugs with once-daily R (10 mg/kg), isoniazid (H) (10 mg/kg), pyrazinamide (Z) (35 mg/kg), and ethambutol (E) (30 mg/kg) (2RHZE/10RHE regimen per index TB guidelines, India) along with pyridoxine (25 mg/day).^4^ At 1-month follow-up, fever and back pain resolved. Serum CRP normalized. MRI of back was unchanged. Currently, the patient being followed up per index TB guidelines, India, with further imaging planned.^4^

Spinal TB represents 2% of all TB cases, 15% of extrapulmonary TB, and 50% of skeletal TB.^5^ Psoas abscess, a complication of spinal TB, can present with pain as an early symptom; the physical finding of a bulging mass as in this case is unusual but important and should be sought.^6^ This case emphasizes the importance of recognizing “red flag” signs for back pain and considering spinal TB in the differential diagnosis. A high index of suspicion for extrapulmonary TB in the absence of typical pulmonary infection is important to forestall complications. Drainage of paravertebral collections is not necessarily essential to the treatment of TB abscesses, but in the present case accelerated clinical recovery and optimized outcome.
